# Silicon Nanoparticles Improve the Shelf Life and Antioxidant Status of *Lilium*

**DOI:** 10.3390/plants10112338

**Published:** 2021-10-29

**Authors:** Juan Francisco Sánchez-Navarro, Yolanda González-García, Adalberto Benavides-Mendoza, América Berenice Morales-Díaz, Susana González-Morales, Gregorio Cadenas-Pliego, María del Socorro García-Guillermo, Antonio Juárez-Maldonado

**Affiliations:** 1 Maestría en Ciencias en Horticultura, Universidad Autónoma Agraria Antonio Narro, Saltillo 25315, CH, Mexico; juansancheznavarro@uadec.edu.mx; 2 Departamento de Botánica, Universidad Autónoma Agraria Antonio Narro, Saltillo 25315, CH, Mexico; yolanda_glezg@hotmail.com; 3 Departamento de Horticultura, Universidad Autónoma Agraria Antonio Narro, Saltillo 25315, CH, Mexico; abenmen@gmail.com; 4 Centro de Investigación y Estudios Avanzados del Instituto Politécnico Nacional Unidad Saltillo, Ramos Arizpe 25900, CH, Mexico; america.morales@cinvestav.edu.mx; 5 Cátedras CONACYT-Departamento de Horticultura, Universidad Autónoma Agraria Antonio Narro, Saltillo 25315, CH, Mexico; qfb_sgm@hotmail.com; 6 Centro de Investigación en Química Aplicada, Saltillo 25294, CH, Mexico; gregorio.cadenas@ciqa.edu.mx; 7 Analytical Chemistry Laboratory, Cinvestav-Saltillo, Ramos Arizpe 25900, CH, Mexico; socorro.garcia@cinvestav.edu.mx

**Keywords:** nanotechnology, enzymatic and non-enzymatic antioxidants, ornamentals, flower quality

## Abstract

The production of ornamentals is an economic activity of great interest, particularly the production of *Lilium*. This plant is very attractive for its color and shapes; however, the quality of its flower and its shelf life can decrease very fast. Therefore, it is of the utmost importance to develop techniques that allow for increasing both flower quality and shelf life. Nanotechnology has allowed for the use of various materials with unique characteristics. These materials can induce a series of positive responses in plants, among which the production of antioxidant compounds stands out. The objective of this study was to determine the impact of the application of silicone nanoparticles (SiO_2_ NPs) on the quality, shelf life, and antioxidant status of *Lilium*. For this, different concentrations of SiO_2_ NPs (0, 200, 400, 600, 800, and 1000 mg L^−1^) were applied in two ways, foliar and soil, as two independent experiments. The contents of enzymatic (superoxide dismutase, glutathione peroxidase, catalase, ascorbate peroxidase, and phenylalanine ammonia lyase) and non-enzymatic (phenols, flavonoids, and glutathione) antioxidant compounds, the mineral content, flower quality, and shelf life were analyzed. The results showed that the application of SiO_2_ NPs through the foliar method induced a greater flowers’ shelf life (up to 21.62% more than the control); greater contents of Mg, P, and S (up to 25.6%, 69.1%, and 113.9%, respectively, compared to the control); more photosynthetic pigment (up to 65.17% of total chlorophyll); more glutathione peroxidase activity (up to 69.9%); more phenols (up to 25.93%); and greater antioxidant capacity as evaluated by the DPPH method (up to 5.18%). The use of SiO_2_ NPs in the production of *Lilium* is a good alternative method to increase flower quality and shelf life.

## 1. Introduction

The lily is a perennial herbaceous bulbous plant belonging to the genus *Lilium* and the family Liliaceae that is native to China and widely cultivated as an ornamental pot, garden, and cut plant [1,2]. It is also used in the pharmaceutical industry and gastronomy in Asia, Europe, and America [2]. This genus is among the most internationally commercialized flower crops [3]. Its form of propagation is generally by means of bulbs, although some species are also propagated by seeds. Its development is divided into four phases: the elongation of the shoots after sowing, beginning of the flower of the apical meristem, flowering, and senescence after flowering [4].

Maintaining flower quality and prolonging shelf life are of great importance, since longevity and quality are economically important factors for lilies’ commercialization due to their short shelf life before or after harvest and deterioration, which cause difficulties for long-distance transport and a decrease in market value [5]. Senescence and poor flower quality are the main reasons for the short shelf life, which is mainly associated with an increase in lipid peroxidation and modifications of the cell wall. It is also due to other factors, such as increases in respiration and activities of hydrolytic enzymes, changes in various cell organelles, the degradation of macromolecules, microbial activities, production and sensitivity to ethylene and other hormones, and oxidative stress by reactive oxygen species (ROS) [6,7].

To overcome these problems, several postharvest technologies have been developed for the global flower industry, many of which involve the use of chemicals to inhibit the synthesis of hormones in the petals or microbial growth in the vessels of the xylem [8]. However, in the last decade, nanotechnology has developed enough to allow for its use in the agricultural industry, where it has proven its effectiveness in increasing production and reducing post-harvest losses, thus leading to a steady increase in application [3].

Silicon is considered a beneficial element for plants, although it is not essential. Moreover, its use has been reported to mitigate different types of stress in plants, mainly due to its accumulation in different plant tissues and the modification of metabolism [9]. In addition, silicon has been shown to be efficient in improving the antioxidant defense systems of plants when they are under conditions of some type of stress [10]. At the nanoscale, silicon may be more efficient at inducing responses in plants due to the new properties of materials at this scale. Therefore, studying the responses of plants with this technology is of great interest.

Silicon nanoparticles (SiO_2_ NPs) have positive effects on the growth, physiology, and protection of plants, since they can significantly improve the processes of water absorption and nutrient supply; positively regulate the processes of photosynthesis and gas exchange; and activate metabolic processes, thus improving the antioxidant defense system and nitrogen metabolism [11,12]. Another positive impact of the application of SiO_2_ NPs is the mitigation of the negative effect of stress by UV radiation, metal toxicity, saline stress, and biotic stress [9]. SiO_2_ NPs can also directly serve as nano-pesticides, nano-herbicides, and nano-fertilizers [13].

The objective of this study was to determine the impact of the application of SiO_2_ NPs in *Lilium* plants—specifically, to determine the best way to apply SiO_2_ NPs, as well as the most suitable concentration to improve flower quality and shelf life.

## 2. Results

### 2.1. Flower Characteristics

**Figure 1:**
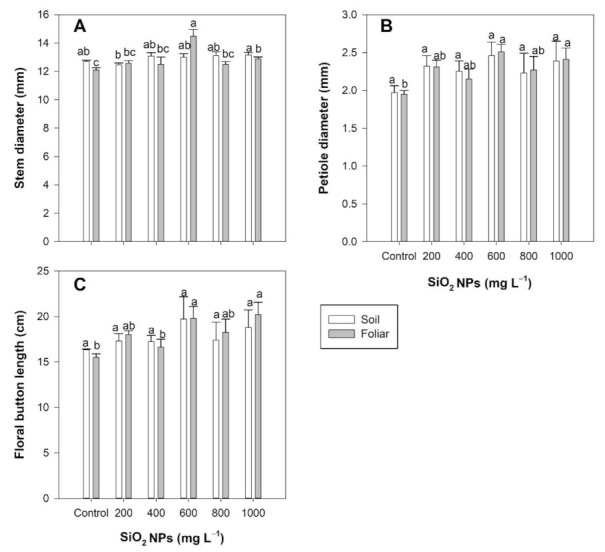
Effect of the application of SiO_2_ NPs on the stem diameter (**A**), petiole diameter (**B**), and button length (**C**) of *Lilium* plants. Different letters indicate significant differences according to Fisher’s least significant difference test (*p* ≤ 0.05).

No differences were observed in stem diameter following the application of SiO_2_ NPs via the soil application method. However, following the foliar application of SiO_2_ NPs, the stem diameter was increased by 19.60% and 6.44% compared to the control with concentrations of 600 and 1000 mg L^−1^, respectively (Figure 1A).

No differences in petiole diameter were observed between treatments when the SiO_2_ NPs were applied by the soil method. However, the foliar application of SiO_2_ NPs induced increases of 28.71% and 23.58% with concentrations of 600 and 1000 mg L^−1^, respectively, compared to the control (Figure 1B).

No differences were observed in the length of the flower bud following application via the soil method. However, with the foliar method, concentrations of 600 and 1000 mg L^−1^ of SiO_2_ NPs showed increases of 27.77% and 30.27%, respectively, compared to the control (Figure 1C).

**Figure 2:**
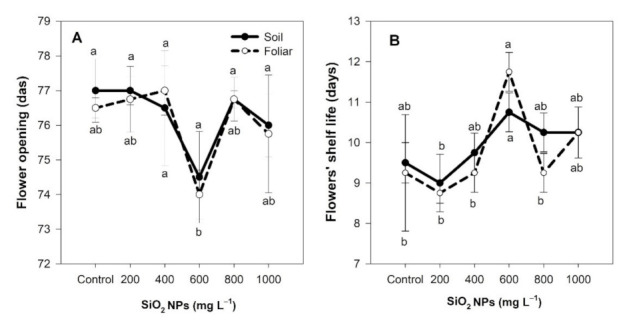
Effect of the application of SiO_2_ NPs on the opening of the flower bud (**A**) and the flowers’ shelf life (**B**). Different letters indicate significant differences according to Fisher’s least significant difference test (*p* ≤ 0.05). das, days after sowing; days, the time (in days) for which the flowers were of good quality.

The opening of the flower bud and the flowers’ shelf life behaved statistically the same when treatments were applied via the soil method. In contrast, when applied via the foliar method, the opening of the flower bud was faster at the concentration of 600 mg L^−1^, although the difference was not significant compared to the control (Figure 2A). The flowers’ shelf life was increased by up to 21.62% more than the control with the application of 600 mg L^−1^ of SiO_2_ NPs (Figure 2B).

### 2.2. Accumulation of Macro- and Micronutrients

The application of SiO_2_ NPs via the soil method modified the concentration of some macronutrients in the leaves, such as Mg, P, and S; however, it had no effect on the macronutrient content in the flowers (Table 1). Consistently, a concentration of 600 mg L^−1^ of SiO_2_ NPs increased the content of Mg, P, and S by 25.6%, 69.1%, and 113.9%, respectively, compared to the control.

Following the application of SiO_2_ NPs via the foliar method, only the calcium content in the flower was modified; the highest concentration, 56.4% more than the control, was observed with 400 mg L^−1^ of SiO_2_ NPs (Table 1).

Regarding micronutrient contents, the application of SiO_2_ NPs via the soil method in concentrations of 600 and 800 mg L^−1^ increased the content of Zn in the leaves by approximately 64% compared to the control. In contrast, the copper content in the flowers decreased with treatments of 400, 800, and 1000 mg L^−1^ of SiO_2_ NPs (Table 2).

The foliar application of SiO_2_ NPs only modified the Zn content in the flowers, as the 400 mg L^−1^ treatment increased the content of this element by 34.1% compared to the control (Table 2).

### 2.3. Photosynthetic Pigments

**Figure 3:**
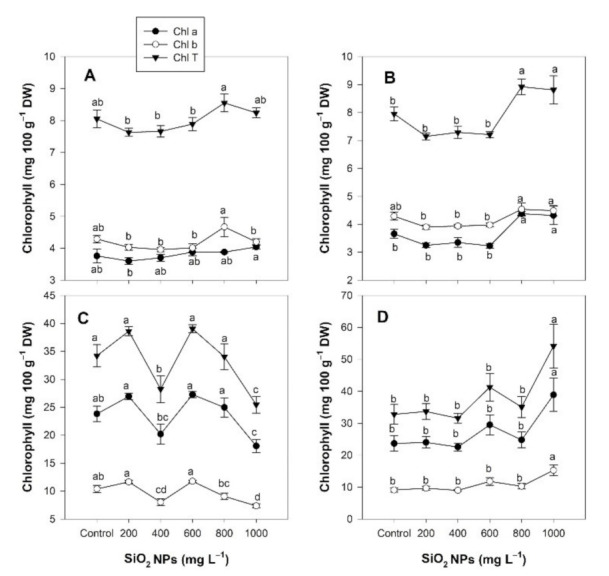
Effect of the application of SiO_2_ NPs via the soil method (**A**,**C**) and the foliar method (**B**,**D**) on the concentration of chlorophyll in the flowers (**A**,**B**) and leaves (**C**,**D**) of *Lilium*. Different letters indicate significant differences according to Fisher’s least significant difference test (*p* ≤ 0.05).

The chlorophyll content in the *Lilium* flowers exposed to different concentrations of SiO_2_ NPs did not show differences between treatments; however, a clear increasing trend was observed with 800 mg L^−1^ of SiO_2_ NPs when the application was carried out via the soil method (Figure 3A). When SiO_2_ NPs were applied through the foliar method, the chlorophyll *a* content increased in the flowers by 19.67% and 18.03% and the total chlorophyll increased by 12.20% and 10.81% with concentrations of 800 and 1000 mg L^−1^, respectively (Figure 3B).

In the leaves, the chlorophyll content decreased following the application of SiO_2_ NPs when they were applied to the soil; chlorophyll *a* decreased by 24.09%, chlorophyll *b* decreased by 29.07%, and total chlorophyll decreased by 25.57% at a concentration of 1000 mg L^−1^ (Figure 3C). Meanwhile, foliar application induced increases in chlorophyll at a concentration of 1000 mg L^−1^ of SiO_2_ NPs: 64.25% in chlorophyll *a*, 67.54% in chlorophyll *b*, and 65.17% in total chlorophyll (Figure 3D).

### 2.4. Antioxidant Enzymes

**Figure 4:**
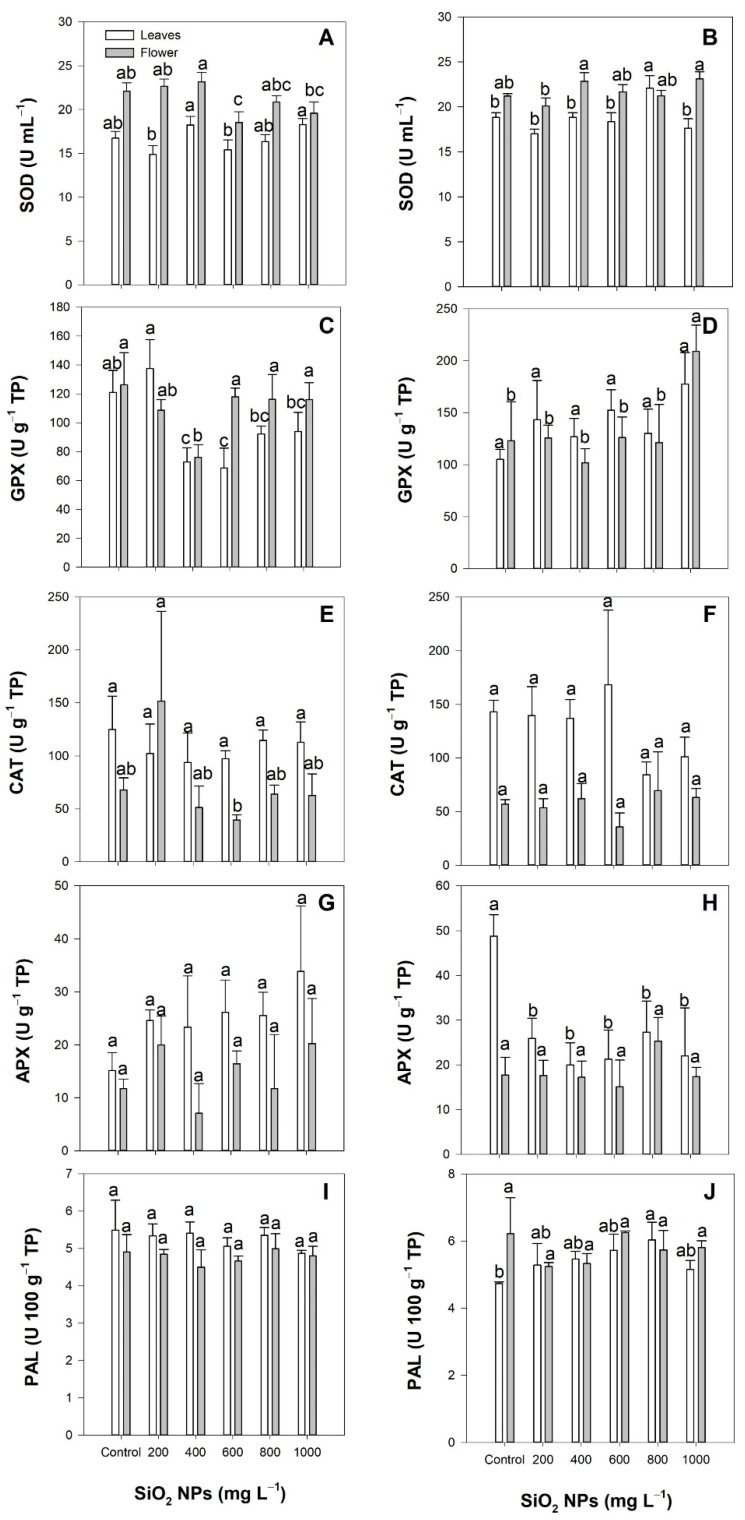
Effect of the application of SiO_2_ NPs via the soil method (**A**,**C**,**E**,**G**,**I**) and the foliar method (**B**,**D**,**F**,**H**,**J**) on the activity of the antioxidant enzymes SOD (**A**,**B**), GPX (**C**,**D**), CAT (**E**,**F**), APX (**G**,**H**), and PAL (**I**,**J**) in *Lilium*. Different letters indicate significant differences according to Fisher’s least significant difference test (*p* ≤ 0.05).

The application of SiO_2_ NPs via the soil method did not modify the activity of superoxide dismutase (SOD) in the leaves compared to the control; only a decrease in the activity of this enzyme was observed ay a concentration of 600 mg L^−1^ in the flowers (Figure 4A). In the case of foliar application, the activity of this enzyme in the flowers was not modified by the application of SiO_2_ NPs; however, an increase of 6.21% was observed in the leaves ay a concentration of 800 mg L^−1^ (Figure 4B).

The glutathione peroxidase (GPX) activity in the leaves decreased by 39.85% and 43.27% ay concentrations of 400 and 600 mg L^−1^, respectively, compared to the control when the SiO_2_ NPs were applied via the soil. In the flowers, the GPX activity also decreased by 39.7%, at a concentration of 400 mg L^−1^ (Figure 4C). Following application through the foliar method, the activity of GPX in the leaves did not show differences between treatments. In contrast, GPX activity increased by 69.9% in the flowers at a concentration of 1000 mg L^−1^ of SiO_2_ NPs compared to the control (Figure 4D).

The catalase (CAT) activity did not show differences between treatments or application methods in either of the two evaluated organs (Figure 4E,F).

The ascorbate peroxidase (APX) activity was not modified when the treatment was applied to the soil, neither in the leaves nor in the flowers (Figure 4G). When the application was through the foliar method, the activity of APX in the leaves decreased in all treatments, while there were no differences between the treatments in the flowers (Figure 4H).

The phenylalanine ammonia lyase (PAL) enzymatic activity did not show differences between treatments, neither in the leaves nor in the flowers, following the application of SiO_2_ NPs via the soil method (Figure 4I). In contrast, the foliar application of 800 mg L^−1^ of SiO_2_ NPs increased the PAL activity in the leaves by 27.48%, while there were no differences between the treatments in the flowers (Figure 4J).

### 2.5. Non-Enzymatic Antioxidant Compounds

**Figure 5:**
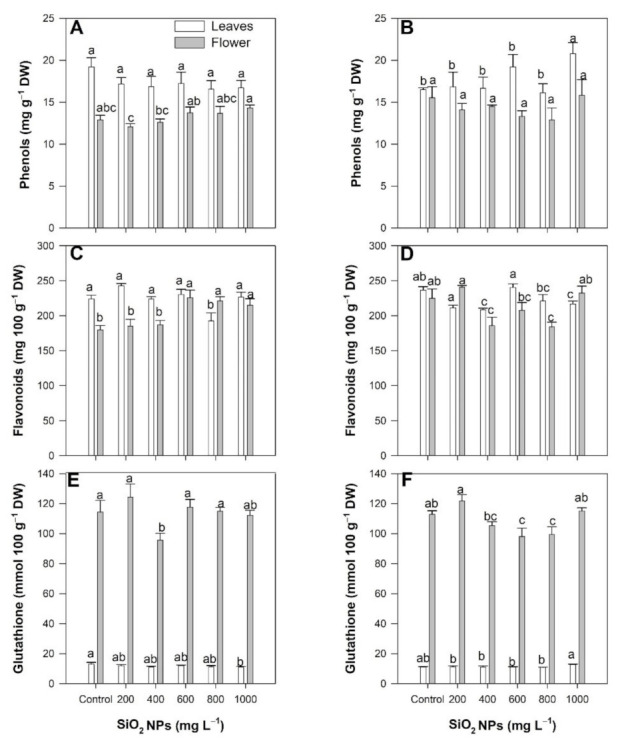
Effect of the application of SiO_2_ NPs via the soil method (**A**,**C**,**E**) and the foliar method (**B**,**D**,**F**) on the non-enzymatic antioxidants: phenols (**A**,**B**), flavonoids (**C**,**D**), and glutathione (**E**,**F**). Different letters indicate significant differences according to Fisher’s least significant difference test (*p* ≤ 0.05).

The phenol content did not show differences between the treatments compared to the control when the SiO_2_ NPs were applied via the soil method in any of the evaluated organs (Figure 5A). When the SiO_2_ NPs were applied via through the foliar method at a concentration of 1000 mg L^−1^, the content of phenols in the leaves increased by 25.93%; however, there were no differences between the treatments in the flowers (Figure 5B).

Following application via the soil method, the flavonoid content in the leaves did not show differences between treatments. However, there were increases of 25.65%, 23.02%, and 19.64% in the flowers at concentrations of 600, 800, and 1000 mg L^−1^ of SiO_2_ NPs, respectively, compared to the control (Figure 5C). In contrast, following the foliar application of SiO_2_ NPs, the flavonoid content in the leaves decreased by 10.59%, 11.86%, and 8.47% at 200, 400, and 800 mg L^−1^ of SiO_2_ NPs, respectively. The flavonoid content in the flowers decreased 17.50% and 18.25% at SiO_2_ NP concentrations of 400 and 800 mg L^−1^, respectively (Figure 5D).

The glutathione content in the leaves decreased by 16% with 1000 mg L^−1^ of SiO_2_ NPs applied via the soil method, and in the flowers, it decreased by 16.67% with the application of 400 mg L^−1^ (Figure 5E). Following foliar application, we observed decreases in glutathione of 13.07% and 11.72% in the flowers at 600 and 800 mg L^−1^ of SiO_2_ NPs, respectively; meanwhile, there were no differences between treatments in the leaves (Figure 5F).

### 2.6. Antioxidant Capacity

**Figure 6:**
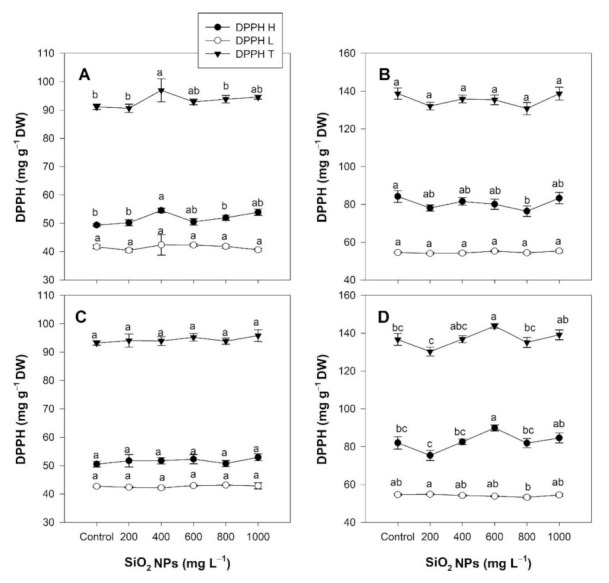
Effect of the application of SiO_2_ NPs via the soil method (**A**,**C**) and the foliar method (**B**,**D**) on the antioxidant capacity in the leaves (**A**,**B**) and flowers (**C**,**D**) of *Lilium* plants. DPPH H: antioxidant capacity of the hydrophilic compounds. DPPH L: antioxidant capacity of the lipophilic compounds. DPPH T: total antioxidant capacity. Different letters indicate significant differences according to Fisher’s least significant difference test (*p* ≤ 0.05).

As evaluated with the 2,2-diphenyl-1-picrylhydrazyl (DPPH) radical, the total antioxidant capacity increased by 6.43% in the leaves following the soil application of 400 mg L^−1^ of SiO_2_ NPs due to the 10.21% increase in the antioxidant capacity of the hydrophilic compounds (Figure 6A). In contrast, a decrease of 9.27% in the antioxidant capacity of hydrophilic compounds was observed in the flowers at 800 mg L^−1^ of SiO_2_ NPs (Figure 6B).

Following foliar application, the antioxidant capacity in the leaves was not modified by the SiO_2_ NPs (Figure 6C). In contrast, the foliar application of SiO_2_ NPs increased the antioxidant capacity of the flowers by 5.18% at a concentration of 600 mg L^−1^ due to the 9.53% increase in the antioxidant capacity of the hydrophilic compounds compared to the control (Figure 6D).

## 3. Discussion

During their development and maturation, flowers require nutrients to keep their metabolism optimal. However, when metabolism is affected, the release of amino acids and monosaccharides from the cell wall can be promoted, since ATP is obtained from them. This leads to the degradation of cell walls and, consequently, to decreases in the life and quality of flowers. Additionally, damage to membranes causes the production of ROS, which results in lipid peroxidation and, ultimately, the senescence of plants [14]. Particularly in the case of cut flowers, senescence is closely linked to the lipid peroxidation of the petals [15]. Thus, an adequate availability of carbohydrates improves the shelf life by acting as a source of energy and nutrition, especially for flower stems with developing buds [16].

Oxidative stress is one of the most important factors that affects both the life of flowers and the quality of cut flowers. Oxidative stress affects cell mechanisms and membrane lipids, accelerating senescence, which is why avoiding or reducing oxidative stress is crucial to increasing the lifespan of flowers [17]. Therefore, an adequate antioxidant defense system can increase the shelf life of flowers, as observed in this study of the application of SiO_2_ NPs.

It has been widely documented that various nanomaterials (NMs) can induce positive responses (such as better growth and development) and changes in metabolism in plants, which can help tolerate different stress conditions [18,19,20,21,22,23]. Nanomaterials have unique properties, including a high surface/volume ratio that results in a high surface energy of these materials; this makes them more reactive, and they can impact plants in different ways when interacting [24]. Interactions can occur from the moment NMs come into contact with a cell, i.e., the cell wall and cell membrane, where a series of signals and changes are produced in the transport of metabolites and ions, as well as the production of ROS; additionally, when they enter the cell, they also interact with different organelles and induce oxidative stress. The results of these interactions can be positive, negative, or null for plants, and they are highly dependent on the amount and type of NMs [25].

NMs activate the antioxidant defense system, and the synthesis of enzymatic and non-enzymatic antioxidant compounds is increased [18,19,20]. The ability of NMs to modify the antioxidant system of plants is one of their main effects, and it is related to the ability to tolerate stress [21]. However, they also have the ability to modify secondary metabolism and, therefore, the synthesis of secondary metabolites such as phenols and flavonoids [22,23].

Silicon is one of the elements that is considered beneficial for plants; when applied, it induces a series of beneficial responses, such as greater growth and development, as well as tolerance to different conditions of both biotic and abiotic stress [26,27,28]. One of the characteristics of silicon that can influence shelf life is that once it penetrates and moves through a plant, it is deposited in the structures where transpiration occurs and becomes insoluble crystals [29]. This makes the cell walls stiffer and, therefore, more durable over time, and it can reduce degradation and extend the shelf life of flowers. In the form of NPs, silicon can increase its ability to induce these effects due to the unique characteristics of nanoscale materials [24], as observed in the present study.

In addition, several studies have shown that the use of silicon-based NPs improves some metabolic processes in plants related to the antioxidant system and, through these, increases the synthesis of antioxidant compounds. In *Dracocephalum kotschyi* plants developed in an MS culture medium and with the application of SiO_2_ NPs, an increase in flavonoids (xanthomicrol, cirsimaritin, and isokaempferide) was observed. This was due to the overexpression of the phenylalanine ammonia lyase and rosmarinic acid synthase (RAS) genes that are closely linked to the rosmarinic acid biosynthetic pathway [30]. The contents of phenols, flavonoids, and tropane alkaloids (hyoscyamine and scopolamine) were also increased in *Hyoscyamus reticulatus* and *H. pusillus* plants in which SiO_2_ NPs were directly applied to the root. In this case, the overexpressed genes were *pmt* (putrescine N-methyltransferase) and *h6h* (hyoscyamine 6 β-hydroxylase) [31]. This showed that the positive effects observed in the antioxidant compounds of *Lilium* may be related to the impact of SiO_2_ NPs on the antioxidant defense system and the secondary metabolism of plants.

SiO_2_ NPs can modify the uptake of nutrients in plants due to different mechanisms, such as the production of organic acids by the roots, that can ultimately facilitate nutrient uptake [32]. Particularly, citric, oxalic, and malic acids form complexes with metals, which affect the fixation, mobility, and availability of nutrients to plants [33]. Moreover, by interacting with the cell walls and membranes of a root, NPs can modify the activity of transporters [34]. Silicon has been shown to have the ability to activate H^+^-ATPases located in the plasma membrane and increase the uptake of potassium through electrochemical gradients, K^+^ channels, and transporters [35].

Le et al. [36] reported a decrease in the concentrations of Cu and Mg in shoots of Bt-transgenic cotton following the application of SiO_2_ NPs. González-Moscoso et al. [37] reported decreases in the Fe concentration in tomato leaves with the application of SiO_2_ NPs; however, they observed increases in the concentrations of Cu and Zn. Tripathi et al. [38] reported increases in K and P in the roots and leaves of *Pisum sativum* seedlings following the addition of Si NPs. Alsaeedi et al. [39] reported increases in the uptake of K in different organs of *Cucumis sativus* plants following the application of Si NPs.

## 4. Materials and Methods

### 4.1. Establishment of Crops

An experiment was established under greenhouse conditions using *Lilium orientalis* “Oriental hybrid, cv Table Dance” plants. The transplant was carried out in 4 L plastic containers, with a perlite/peat moss mixture in a 1:1 ratio. A directed irrigation system and a 25% concentration Steiner nutrient solution were used [40] to provide the necessary nutrients to the plant. The pH of the solution was adjusted to 6.5, and the electrical conductivity (EC) reached 1.1 dS m^−1^.

### 4.2. Treatments

The treatments consisted of suspensions of SiO_2_ NPs in concentrations of 200, 400, 600, 800, and 1000 mg L^−1^, in addition to a control. These treatments were selected on the basis of the results obtained by Pinedo-Guerrero et al. [41]. Moreover, the study of these authors showed that silicon at the nanoscale (SiO_2_ NPs) is better than ionic silicon (K_2_SiO_3_) at inducing positive responses in plants [41]. SiO_2_ NPs were of 10–20 nm in size, had a spherical morphology, a surface area of 160 m^2^ g^−1^, and a bulk density of 0.08–0.1 g cm^−3^ (SkySpring Nanomaterials Inc., Houston, TX, USA). The application of SiO_2_ NPs was accomplished through two methods, one foliar and the other soil, and each application method formed an independent experiment. A total of five applications were made, starting 15 days after sowing the bulbs (das). In order to conduct an adequate comparison between both application methods, we first applied the foliar treatments and quantified the amount of SiO_2_ NPs in solution so that we could apply the same amount SiO_2_ NPs in solution via soil. Volumes of 2, 4, 6, 8, and 10 mL per plant were used at 15, 29, 43, 57, and 71 days, respectively, for both methods of application.

### 4.3. Agronomic Evaluations

To determine the effect of the treatments on the development of the plants and flowers, the diameter of the plant stems, the diameter of the flower stalks, and the size of the flowers were determined. The stem diameters were determined between the second and third leaves of the plants. The diameters of the flower stalks were determined at the junction with the flower calyx. The sizes of the flowers were determined prior to opening from the calyx to the apex of the flowers. In addition, we quantified both the days since sowing to the opening of the flower buds and the shelf life of the plants (from the opening of the first flower bud to the loss of the first flower petal).

### 4.4. Macro- and Micronutrient Determination

The contents of macronutrients (Ca, Mg, P, and S), micronutrients (Fe, Mn, Zn, and Cu), and silicon in leaves and flowers were determined with a plasma emission spectrophotometer (Optima 8300 ICP-OES Optical System, PerkinElmer, MA, USA). For this, dry tissue of leaves and flowers (1 g of each sample) was taken and digested in 30 mL of nitric acid at 300 °C over a time period of 6 h. After this, the volume of each sample was brought to 50 mL with deionized water for analysis.

### 4.5. Biochemical Analysis

#### 4.5.1. Photosynthetic Pigments

Chlorophyll a, chlorophyll b, and total chlorophyll were determined using 0.1 g of lyophilized tissue to which 20 mL of a hexane/acetone solution (3:2) were added. These samples were centrifuged, and aliquots were taken from the supernatants and measured in a UV–vis spectrophotometer (UNICO Model UV2150, Dayton, NJ, USA) at 645 and 663 nm. The chlorophyll concentration (mg 100 g^−1^ dry weight) was calculated using the equations described by Nagata and Yamashita [42].

#### 4.5.2. Antioxidant Enzymes

Prior to the analysis, we obtained an extract with which the enzymes were determined. Then, 200 mg of previously lyophilized leaf and flower plant tissue and 20 mg of polyvinyl pyrrolidone were used. This mixture was extracted by adding 1.5 mL of phosphate buffer at pH 7 (0.1 M), sonicating for 5 min, and centrifuging in a centrifuge (OHAUS Frontier Model FC5515 R, Parsippany, NJ, USA) at 17,500× *g* for 10 min at 4 °C. The supernatant was collected and filtered with a 0.45 micron pore PTFE membrane. Finally, it was diluted to 1:20 with phosphate buffer at pH 7 (0.1 M). With this extract, we determined the different enzymes, and we determined the total protein content (TP, mg g^−1^ DW) with Bradford’s colorimetric technique [43]. In a microplate, 5 µL of the extract and 250 µL of the Bradford reagent were placed in each well. This mixture was incubated for 10 min at room temperature (26 °C) and then read at a 630 nm wavelength in a microplate reader (Allsheng, model AMR-100, Hangzhou, China). The total proteins were used to calculate the specific activity of each enzyme.

Superoxide dismutase (SOD) (EC 1.15.1.1) was evaluated out using a 706,002 SOD Cayman^®^ Kit plus 20 μL of enzymatic extract; the absorbance was measured at a length of 450 nm using a plate reader (Allsheng, model AMR-100, Hangzhou, China). The results are expressed as units per gram of total proteins (U mL^−1^), where U is defined as the amount of enzyme necessary to exhibit at 50% dismutation of the superoxide radical.

Catalase (CAT) (EC 1.11.1.6) was quantified by the spectrophotometric method used by Dhindsa et al. [44]. The measurement was carried out twice (at time 0 (T0) and at time 1 (T1)). At T0, 100 μL of the enzyme extract, 400 μL of H_2_SO_4_ (5%), and 1000 μL of H_2_O_2_ (100 mM) were added to a test tube and mixed. Then, the absorbance was measured at 270 nm in a UV–vis spectrophotometer (UNICO Model UV2150, Dayton, NJ, USA) using a quartz cell. The measurement at T1 was taken after 60 s of reaction. The difference between T1 and T0 was used to obtain the activity of the enzyme. The results are expressed as U g^−1^ TP, where U is equal to the millimole equivalent of H_2_O_2_ consumed per milliliter per minute.

Ascorbate peroxidase (APX) (EC 1.11.1.11) was evaluated in accordance with the method of Nakano and Asada [45]. The measurement was performed twice (at time 0 (T0) and at time 1 (T1)). At T0, a mixture of 100 µL of the enzyme extract, 500 µL of ascorbate (10 mg L^−1^), 400 µL of H_2_SO_4_ (5%), and 1000 µL of H_2_O_2_ (100 mM) was placed in a test tube and then mixed. The absorbance at 266 nm was measured in a UV–vis spectrophotometer (UNICO Model UV2150, Dayton, NJ, USA) using a quartz cell. The measurement at T1 was taken after 60 s of reaction. The difference between T1 and T0 was used to obtain the activity of the enzyme. The results are expressed as U g^−1^ TP, where U is equal to one micromole of oxidized ascorbate per milliliter per minute.

Glutathione peroxidase (GPX) (EC 1.11.1.9) was determined with the method of Xue et al. [46]. First, 200 μL of the enzyme extract, 400 μL of reduced glutathione (GSH, 0.1 mM), and 200 μL of Na_2_HPO_4_ (0.067 M) were mixed in a tube. The mixture was preheated in a water bath at 25 °C for 5 min, and then 200 µL of H_2_O_2_ (1.3 mM) were added to start the catalytic reaction for 10 min at a temperature of 26 °C. The reaction was stopped by adding 1000 µL of 1% trichloroacetic acid. The extraction was placed in an ice bath for 30 min and centrifuged at 1000× *g* for 10 min at 4 °C. To evaluate glutathione peroxidase, 480 μL of the supernatant, 1200 μL of Na_2_HPO_4_ (0.32 M), and 320 μL of 1 mM 5,5-dithio-bis-2-nitrobenzoic acid (DTNB) were mixed in a tube. The absorbance at 412 nm was measured using a UV–vis spectrophotometer (UNICO Model UV2150 Spectrophotometer, Dayton, NJ, USA) using a quartz cell. The results are expressed as U g^−1^ TP, where U is equal to the millimole equivalent of reduced glutathione (GSH) per milliliter per minute.

Phenylalanine ammonia lyase (PAL) (EC 4.3.1.5) was determined according to the method of Sykłowska-Baranek et al. [47]. An extraction was carried out by mixing 100 µL of the enzyme extract and 900 µL of L-phenylalanine (6 mM), incubating the mixture at 40 °C for 30 min, and then stopping the reaction by adding 250 µL of HCl (5 N). The samples were placed in a bath of ice, and 750 µL of distilled water were added. The absorbance at 290 nm was measured in a UV–vis spectrophotometer (UNICO Model UV2150 Spectrophotometer, Dayton, NJ, USA) using a quartz cell. The results are expressed as U g^−1^ TP, where U is equal to the micromole equivalent of trans-cinnamic acid per milliliter per minute.

#### 4.5.3. Non-Enzymatic Antioxidant Compounds and Antioxidant Capacity

Phenols, expressed as milligrams per gram of dry weight (mg g^−1^ DW), were determined using the Folin–Ciocalteu reagent, as described by Singleton et al. [48]. First, 200 mg of lyophilized tissue were extracted by adding 1000 µL of a water/acetone solution (1:1). The mixture was stirred for 30 s and centrifuged in a centrifuge (OHAUS Frontier Model FC5515 R, Parsippany, NJ, USA) at 17,500× *g* for 10 min at 4 °C. To evaluate the phenolic compounds, 18 μL of the supernatant, 70 μL of Folin–Ciocalteu reagent (0.2 N), 175 µL of 20% sodium carbonate (Na_2_CO_3_), and 1740 mL of distilled water were mixed. Then, the mixture was placed in a homogenization vortex for 30 s before being placed in a 45 °C water bath for 30 min. Finally, the reading was taken at an absorbance of 750 nm using a quartz cell in a UV–vis spectrophotometer (UNICO Model UV2150 Spectrophotometer, Dayton, NJ, USA).

Flavonoids (mg 100 g^−1^ DW) were evaluated with the method of Arvouet-Grand et al. [49]. For extraction, 20 mg of lyophilized tissue were placed in a test tube, where 2000 µL of reagent-grade methanol were added and stirred for 30 until the mixture was homogenized. The mixture was filtered on No. 1 Whatman paper. For quantification, 2000 µL of the extract and 2000 µL of 2% methanolic aluminum trichloride (AlCl_3_) solution were added to a test tube and allowed to stand for 20 min in the dark. The reading was then taken in a UV–vis spectrophotometer (UNICO Model UV2150 Spectrophotometer, Dayton, NJ, USA) at a wavelength of 415 nm using a quartz cell.

Glutathione (mmol 100 g^−1^ DW) was determined using the method of Xue et al. [46] by means of a 5,5-dithio-bis-2 nitrobenzoic acid (DTNB) reaction. A mixture of 0.480 mL of the extract, 2.2 mL of sodium dibasic phosphate (Na_2_HPO_4_ at 0.32 M), and 0.32 mL of the DTNB dye (1 mM) was placed in a test tube. Then, the mixture was vortexed and read on a UV–Vis spectrophotometer (UNICO Model UV2150 Spectrophotometer, Dayton, NJ, USA) at 412 nm using a quartz cell.

The antioxidant capacity was determined using the DPPH (2,2-diphenyl-1-picrylhydrazyl) radical according to the method of Brand-Williams et al. [50]. The hydrophilic compounds were determined using the enzymatic extract with a phosphate buffer at pH 7 (0.1 M), and a hexane/acetone extracting solution was used for the lipophilic compounds. The total antioxidant capacity was obtained by the sum of hydrophilic and lipophilic compounds [51]. The antioxidant capacity was expressed as equivalent vitamin C (mg g^−1^ DW).

### 4.6. Statistical Analysis

The experiments were established in a randomized complete block design, and four replications per treatment were considered. An analysis of variance and a mean comparison test were performed with Fisher’s least significant difference method (*p* ≤ 0.05). The entire process was carried out using the Infostat software (2020).

## 5. Conclusions

The use of SiO_2_ NPs in the production of *Lilium* proved to be an effective alternative to improve the shelf life of flowers. However, it is important to consider the route of application, as the results of our chosen routes were different. In this study, it was consistently observed that the foliar application of SiO_2_ NPs was more effective than the soil application in improving the antioxidant system of plants, which resulted in a longer shelf life. The results showed a greater shelf life of the flowers; greater contents of Mg, P, and S; more photosynthetic pigments; and greater glutathione peroxidase, phenols, and antioxidant activity. All of these positive impacts can increase the quality of flowers.

Therefore, implementing the use of SiO_2_ NPs in the production of flowers, such as *Lilium* and other species of commercial interest, may be an excellent option to improve the quality of flowers and extend their shelf life.

## Figures and Tables

**Table 1:** Effect of the application of SiO_2_ NPs on the concentration of macronutrients in leaves and flowers.

		Leaves				Flowers			
	Treatment	Ca	Mg	P	S	Ca	Mg	P	S
Soil	Control	1.04 ± 0.09 a	1.60 ± 0.11 bc	2.30 ± 0.08 c	0.77 ± 0.06 d	12.39 ± 0.61 a	4.70 ± 0.21 a	2.79 ± 0.17 a	1.33 ± 0.06 a
	200	0.67 ± 0.36 a	1.49 ± 0.09 c	2.98 ± 0.24 bc	0.90 ± 0.13 cd	13.94 ± 0.50 a	4.24 ± 0.07 a	3.52 ± 0.29 a	1.61 ± 0.06 a
	400	0.68 ± 0.14 a	1.89 ± 0.08 ab	3.43 ± 0.07 ab	1.26 ± 0.05 b	13.59 ± 1.21 a	4.20 ± 0.30 a	3.32 ± 0.13 a	1.55 ± 0.06 a
	600	0.78 ± 0.23 a	2.01 ± 0.07 a	3.89 ± 0.27 a	1.64 ± 0.06 a	15.23 ± 1.12 a	4.27 ± 0.20 a	3.18 ± 0.30 a	1.69 ± 0.21 a
	800	0.58 ± 0.05 a	1.59 ± 0.14 c	3.11 ± 0.40 b	1.10 ± 0.14 bc	11.99 ± 1.78 a	3.76 ± 0.41 a	3.18 ± 0.34 a	1.48 ± 0.19 a
	1000	0.56 ± 0.13 a	1.78 ± 0.09 abc	3.46 ± 0.16 ab	1.33 ± 0.09 b	12.78 ± 1.56 a	4.33 ± 0.55 a	3.05 ± 0.43 a	1.53 ± 0.15 a
	*p*-value	NS	0.0125	0.0033	0.0010	NS	NS	NS	NS
Foliar	Control	1.08 ± 0.21 a	1.91 ± 0.16 a	3.68 ± 0.33 a	1.32 ± 0.13 a	9.72 ± 1.56 c	3.74 ± 0.67 a	2.75 ± 0.17 a	1.09 ± 0.19 a
	200	0.80 ± 0.17 a	1.89 ± 0.07 a	3.27 ± 0.10 a	1.26 ± 0.02a	14.2 ± 0.51 ab	4.67 ± 0.20 a	2.85 ± 0.35 a	1.44 ± 0.08 a
	400	0.47 ± 0.06 a	1.85 ± 0.07 a	3.59 ± 0.16 a	1.24 ± 0.03 a	15.2 ± 0.82 a	4.89 ± 0.23 a	3.45 ± 0.26 a	1.54 ± 0.06 a
	600	0.72 ± 0.18 a	1.86 ± 0.24 a	3.52 ± 0.38 a	1.20 ± 0.11 a	12.53 ± 1.49 abc	4.24 ± 0.50 a	2.83 ± 0.33 a	1.37 ± 0.22 a
	800	0.91 ± 0.12 a	1.71 ± 0.05 a	3.28 ± 0.23 a	1.28 ± 0.07 a	13.91 ± 0.87 ab	4.81 ± 0.30 a	3.3 5 ± 0.22 a	1.62 ± 0.14 a
	1000	0.99 ± 0.22 a	1.89 ± 0.07 a	3.48 ± 0.33 a	1.40 ± 0.06 a	11.13 ± 1.21 bc	3.79 ± 0.30 a	2.92 ± 0.15 a	1.41 ± 0.07 a
	*p*-value	NS	NS	NS	NS	0.0280	NS	NS	NS

Different letters per column indicate significant differences according to Fisher’s least significant difference test (*p* ≤ 0.05). *n* = 4 ± standard error.

**Table 2:** Effect of the application of SiO_2_ NPs on the concentration of micronutrients in leaves and flowers.

		Leaf					Flower				
	Treatment	Fe	Mn	Zn	Cu	Si	Fe	Mn	Zn	Cu	Si
Soil	Control	58.6 ± 4.3 a	13.7 ± 0.8 a	28.5 ± 1.9 c	5.84 ± 1.2 a	41.4 ± 3.7 a	136.6 ± 17.6 a	35.9 ± 2.6 a	32.6 ± 2.3 a	7.83 ± 0.6 a	45.5 ± 1.4 a
	200	58.1 ± 9.0 a	13.8 ± 1.7 a	33.2 ± 4.5 bc	4.76 ± 1.2 a	37.7 ± 8.1 a	93.7 ± 5.0 a	34.5 ± 2.5 a	33.9 ± 3.8 a	6.46 ± 0.3 ab	57.4 ± 10.0 a
	400	51.8 ± 3.3 a	14.7 ± 0.8 a	36.1 ± 2.8 abc	3.98 ± 0.3 a	29.8 ± 2.2 a	110.7 ± 5.7 a	30.3 ± 3.5 a	29.5 ± 1.6 a	5.44 ± 0.6 b	37.2 ± 1.8 a
	600	63.2 ± 9.8 a	15.9 ± 0.9 a	47.4 ± 1.5 a	5.20 ± 0.3 a	31.7 ± 3.4 a	119.3 ± 6.1 a	36.4 ± 1.0 a	36.6 ± 1.8 a	7.99 ± 1.5 a	40.7 ± 2.6 a
	800	99.5 ± 26.1 a	13.2 ± 1.4 a	46.6 ± 7.3 a	3.81 ± 0.8 a	33.2 ± 2.7 a	110.5 ± 5.3 a	28.1 ± 4.1 a	33.9 ± 3.1 a	5.10 ± 0.8 b	42.0 ± 0.6 a
	1000	76.4 ± 11.5 a	13.8 ± 0.4 a	43.3 ± 1.0 ab	3.70 ± 0.2 a	38.5 ± 3.5 a	106.1 ± 8.4 a	29.9 ± 3.3 a	29.4 ± 2.4 a	4.99 ± 0.5 b	38.3 ± 3.0 a
	*p*-value	NS	NS	0.0121	NS	NS	NS	NS	NS	0.0431	NS
Foliar	Control	80.5 ± 3.8 a	15.0 ± 1.2 a	58.5 ± 1.5 a	4.69 ± 0.5 a	34.3 ± 0.4 a	110.1 ± 10.6 a	22.2 ± 2.5 a	33.1 ± 1.1 b	5.39 ± 0.9 a	39.2 ± 1.5 a
	200	122.2 ± 15.6 a	14.2 ± 0.9 a	50.7 ± 1.9 a	4.01 ± 0.4 a	34.9 ± 1.1 a	143.2 ± 5.8 a	28.8 ± 2.5 a	37.3 ± 3.8 ab	4.99 ± 0.4 a	45.4 ± 2.0 a
	400	97.5 ± 12.9 a	14.6 ± 0.8 a	49.7 ± 3.2 a	3.43 ± 0.4 a	36.8 ± 1.5 a	138.1 ± 8.8 a	32.6 ± 2.4 a	44.4 ± 2.9 a	6.85 ± 0.8 a	45.0 ± 7.1 a
	600	88.2 ± 10.4 a	14.2 ± 1.5 a	44.7 ± 6.5 a	3.20 ± 0.3 a	31.2 ± 4.7 a	132.2 ± 16.9 a	27.3 ± 3.0 a	32.6 ± 3.8 b	4.26 ± 0.5 a	28.9 ± 4.2 a
	800	100.1 ± 9.6 a	14.3 ± 1.0 a	48.3 ± 3.4 a	3.11 ± 0.4 a	35.6 ± 3.5 a	144.9 ± 12.3 a	31.3 ± 3.9 a	39.3 ± 1.5 ab	5.59 ± 0.7 a	47.7 ± 10.2 a
	1000	101.3 ± 40.3 a	14.9 ± 0.5 a	50.2 ± 5.3 a	3.43 ± 0.3 a	31.7 ± 3.5 a	174.2 ± 44.2 a	26.2 ± 3.1 a	35.1 ± 0.9 b	4.39 ± 0.3 a	33.3 ± 2.3 a
	*p*-value	NS	NS	NS	NS	NS	NS	NS	0.0479	NS	NS

Different letters per column indicate significant differences according to Fisher’s least significant difference test (*p* ≤ 0.05). *n* = 4 ± standard error.

## Data Availability

Not applicable.
